# Gender Differences in the Association between Positive Drinking Attitudes and Alcohol-Related Problems. The WIRUS Study

**DOI:** 10.3390/ijerph17165949

**Published:** 2020-08-16

**Authors:** Neda S. Hashemi, Mikkel Magnus Thørrisen, Jens Christoffer Skogen, Hildegunn Sagvaag, David Gimeno Ruiz de Porras, Randi Wågø Aas

**Affiliations:** 1 Department of Public Health, Faculty of Health Sciences, University of Stavanger, 4036 Stavanger, Norway; mikkel.m.thorrisen@uis.no (M.M.T.); Jens.Christoffer.Skogen@fhi.no (J.C.S.); hildegunn.sagvaag@uis.no (H.S.); 2 Department of Occupational Therapy, Prosthetics and Orthotics, Faculty of Health Sciences, OsloMet—Oslo Metropolitan University, 0130 Oslo, Norway; 3 Department of Health Promotion, Norwegian Institute of Public Health, 5015 Bergen, Norway; 4 Alcohol & Drug Research Western Norway, Stavanger University Hospital, 4010 Stavanger, Norway; 5 Southwest Center for Occupational and Environmental Health, Department of Epidemiology, Human Genetics, and Environmental Sciences, School of Public Health in San Antonio, The University of Texas Health Science at Houston, Houston, TX 77229, USA; david.gimeno@uth.tmc.edu; 6 Center for Research in Occupational Health (CiSAL), Universitat Pompeu Fabra, 08002 Barcelona, Spain; 7 CIBER of Epidemiology and Public Health, 28029 Madrid, Spain

**Keywords:** alcohol attitudes, norms, gender differences, public health, occupational health, workplace interventions, sick leave, presenteeism

## Abstract

*Background:* Alcohol consumption is deeply integrated in people’s social- and work lives and, thus, constitutes a serious public health challenge. Attitudes toward drinking stand out as important predictors of drinking, but have to date been sparsely studied in employee populations. This study explores the association of employees’ attitudes toward drinking with their alcohol-related problems, and whether this association is moderated by gender and employment sector. *Methods:* Cross-sectional data were collected from a heterogeneous sample of employees (*N* = 4094) at 19 Norwegian companies. Drinking attitudes were assessed using the Drinking Norms Scale. The AUDIT (Alcohol Use Disorders Identification Test) scale was then used to assess any alcohol-related problems. Data were analyzed using chi-square tests, analysis of covariance (ANCOVA), and multiple logistic regression. *Results:* Employees with predominantly positive drinking attitudes were almost three times as likely to report alcohol-related problems compared to employees with more negative drinking attitudes (OR = 2.75; 95% CI: 2.00–3.76). Gender moderated the association between positive drinking attitudes and alcohol-related problems (OR = 3.30; 95% CI: 2.10–5.21). The association was stronger in women (OR = 5.21; 95% CI: 3.34–8.15) than in men (OR = 3.10; 95% CI: 2.11–4.55). Employment sector did not moderate the association between drinking attitudes and alcohol-related problems. *Conclusions:* Employee attitudes toward alcohol should be monitored to better enable early workplace health promotion interventions targeting alcohol problems. These interventions might need to be gender-specific.

## 1. Introduction

Alcohol consumption is deeply integrated in social and work life in many societies [1], and thus constitutes a major public health challenge. A recent study by the Global Burden of Disease Project suggests that alcohol-related consequences are more severe than previously assumed with alcohol consumption being a leading risk factor for mortality and disability-adjusted life years (DALYs) in the global population aged 15 to 49 years [2]. In this age group, approximately 12% of deaths in men and 4% in women can be attributed to alcohol consumption [1]. Risky drinking, i.e., a drinking pattern that raises the likelihood of medical, social, occupational, and economic problems [3], may have adverse consequences on people’s lives, the health care system, workplace productivity, and global economic burden [4,5]. Therefore, reducing harmful drinking is a key issue to ensure greater personal and economic well-being [1,6].

Several authors have emphasized that alcohol consumption in work-related settings can help facilitate efforts for teambuilding and bonding with clients [7,8]. On the other hand, employees’ alcohol consumption is also associated with productivity decrements, such as absenteeism [9] and presenteeism (i.e., reduced on-the-job performance) [10]. Given that the majority of adults spend considerable time at work [11] and that the majority of workers consume alcohol regularly [12,13], there may be a large percentage of employees characterized as risky drinkers who could benefit from preventive interventions [14]. And the workplace may be an ideal setting for such interventions [15].

Alcohol consumption may not be same for all groups of workers, suggesting that intervention may need to be specific for different target groups. For instance, gender differences in alcohol consumption have been previously reported [16,17,18] indicating that men drink more frequently and more heavily than women, while women are overrepresented among abstainers [18]. Hence, due to a dose-response relationship between alcohol consumption and alcohol-related problems [19], men are more prone than women to experiencing alcohol-related problems [17]. This finding may indicate endogenous gender differences, and yet, gender-specific drinking patterns may also be heavily influenced by sociocultural factors. For instance, the magnitude of gender differences in consumption is not consistent by country [18]. Countries with higher gender equality (e.g., the Nordic countries) tend to have smaller gender discrepancies in drinking patterns than countries with lower gender equality [20,21]. For example, in Norway, a drinking pattern convergence between the genders has been observed such as women’s drinking levels has gradually moved toward that of men [22].

Individuals are never totally isolated from their sociocultural surroundings. Sociocultural structures can affect drinking, also affecting gender differences in drinking, and the processes of internalizing social and cultural norms [23]. Drinking cultures exist on different levels (e.g., on national and workplace levels) and generally prescribe what is considered to be appropriate consumption levels, the purposes for drinking and its settings, how to behave during drinking situations, and how to appraise and evaluate different alcohol-related phenomena [7,24,25]. Thus, each culture influences its own alcohol-related perceptions and attitudes differently [26,27,28]. In addition, the distinction between “wet” and “dry” drinking cultures [29] also constitutes a framework that can be influential when understanding drinking cultures. “Wet” cultures are characterized by frequent drinking, high total per capita total consumption, but yet a quite low prevalence of heavy drinking. In contrast, “dry” cultures tend to frequent drinking and lower total per capita consumption, but still a markedly higher occurrence of heavy/binge drinking. At the workplace level, an organization’s drinking culture (i.e., organized set of shared values and understandings about alcohol consumption) may impact the drinking level of its workers [30,31,32,33]. Drinking cultures may vary by work organization and occupation [34], with each occupational culture holding its own structure (e.g., formal and informal), social organization, norms, rituals, history, and beliefs [25,34]. For example, Ames, Grube and Moore studied the same occupational group within two large manufacturing plants showing that differences in internal organizational cultures can considerably affect workers’ attitudes towards drinking with one of the workplaces reporting a more positive attitude towards alcohol drinking than its counterpart [30]. Further, a 2019 report from the Norwegian Institute of Public Health found notable differences in private- versus public-sector employees in Norway with private-sector employees reporting more alcohol intake, more alcohol-related problems, and more positive attitudes towards alcohol than public-sector employees [35]. Moreover, some studies indicated a notable attitude-drinking relationship among employees in different occupations [34,36].

These prior findings stress the importance of sociocultural norms and the related perceptions and attitudes in regards to modifying alcohol-related behaviors. Thus, sociocultural norms prescribe what is considered appropriate in a certain situation [23], subjective norms reflect individuals’ perceptions of these sociocultural prescriptions, and certain attitudes may be considered even more idiosyncratic and all together comprises individuals’ evaluations or appraisals of a certain behavior [37]. One may also assume that “the more favorable the attitude and subjective norm with respect to a behavior, and the greater the perceived behavioral control, the stronger should be an individual’s intention to perform the behavior” ([37], p. 188). The crucial term here is “attitude”, which is a key component of major health behavior theories [38,39,40]. Indeed, attitudes have been identified as potent predictors of drinking quantity, getting drunk, and choosing binge drinking [41]. Individuals who have positive attitudes toward alcohol tend to drink more than individuals who have more negative drinking attitudes [42,43,44,45,46,47]. The relative importance of attitudes when predicting behavior may also vary according to gender. Men and women may hold different attitudes, and the association between their attitudes and their behavior may also be different. Although some studies have explored gender differences in the extent to which drinking attitudes predict drinking behaviors, the results have been inconclusive. Whereas some studies indicated a stronger attitude-drinking relationship among women [41,48], others found just the opposite [49,50,51].

Knowledge of the different associations between drinking attitudes and alcohol-related consequences among adult workers, and whether such associations differ by gender, is important to better understand and prevent alcohol-related problems in the workforce. Adults (age 18 and above) are found to be proper subjects for assessing such attitudes, due to their having more experience with alcohol [41,52,53]. This knowledge may be pertinent when designing and evaluating workplace health promotion programs. Although the existing evidence of an association between drinking attitudes and alcohol consumption in non-work settings is rather robust, that evidence may not be readily applicable to workplace settings for the following reasons: (i) there is a lack of research examining working samples as opposed to college students, which have been predominant in the prior literature [42,43,47,48]; (ii) there are no recent studies; (iii) drinking attitudes have been measured using non-validated items rather than validated instruments, or have measured alcohol consumption in combination with other substance use behaviors [54,55]; and, (iv) examining whether the association between drinking attitudes and alcohol-related problems in workers is moderated by gender and/or employment sector have been not explored in detail. Critically, although previous studies among college students could have some applicability to working populations, findings from those studies could be biased by student peers’ risky behaviors, which have been found to be driven by these individuals’ (mis)perception of their peers’ behavior, regardless of how accurate those perceptions are [56,57,58]. Students normally overestimate the actual drinks as well as the amount of approved alcohol use by others and do not display their real attitudes [43,59,60]. Although adult workers may not be free of such (mis)perceptions, younger populations, like college students, could be more affected by it than older individuals due to not being completely aware of their peer’s normal consumption patterns [61,62,63]. Using a heterogeneous adult working sample and internationally validated instruments, the present study intended to extend the existing literature. 

### Study Aim

The aims of this study were to explore the association between employees’ positive or negative drinking attitudes and alcohol-related problems, and whether this association is moderated by gender and/or employment sector.

## 2. Materials and Methods 

### 2.1. Design

This study is part of the Norwegian national Workplace Interventions Preventing Risky Alcohol Use and Sick Leave (WIRUS) project and was designed as a cross-sectional study of employees in 19 companies in Norway.

### 2.2. Sample and Data Collection

Employees were recruited between 2014 and 2019 from private (*n* = 7) and public (*n* = 12) companies in Norway. The recruitment strategy sought to gather a heterogeneous sample of employees and workplaces. Hence, the 19 companies were recruited based on geographical, sector and industry diversity, representing the following economic activities: Transportation/storage, education, manufacturing, public administration, human health/social work activities, and accommodation/food service. Individual-level criteria for inclusion were: (i) age 16–72; (ii) employee status (salaried-employees in any blue, white or pink-collar occupations); (iii) basic understanding of the Norwegian language; and, (iv) provided written informed consent. 

All employees in the 19 companies (*n* = 17,855) were invited to participate via their employer-provided e-mail address. Altogether, 5076 employees (28.5%) agreed to participate. However, only those participants who responded to all items (*n*= 4094) were included in the current analyses. As shown in Table 1, the sample was predominantly female (*n* = 2696; 65.9%), more than two-thirds were age 40 or older, and 70% had completed a university/college education. Men, when compared to women, were somewhat older, more likely to have primary/lower secondary education as their highest educational attainment, more likely to have a full-time position, and less likely to be employed in the public sector (all *p* < 0.001). Comparisons between the study sample and the invited sample (all eligible employees in the 19 selected companies) revealed a somewhat higher proportion of employees age ≥40 in the study sample (68.9% versus 64.1%), but showed no significant difference in gender distribution.

### 2.3. Measurements

#### 2.3.1. Drinking Attitudes

Drinking attitudes were measured using the Drinking Norms Scale (DNS) [31], a 7-item scale focused on attitudes toward drinking in general (three items) and work-related drinking (four items). Responses were coded on a 4-point Likert scale (1 = strongly disagree; 2 = disagree; 3 = agree; 4 = strongly agree). The seven DNS items demonstrated acceptable internal consistency (Cronbach’s *α* = 0.71). For descriptive analyses, item scores were dichotomized to distinguish between respondents who disagreed (scores 1/2) and those who agreed (scores 3/4) with the statement. To compute the DNS summary scale, negatively worded items (i.e., items 6 and 7) were reversed scored, and a mean score for all seven items was calculated so that the higher score the more positive/liberal drinking attitudes. For the analyses, the mean score was dichotomized based on a median split into “predominantly negative drinking attitudes” (scores < 2.14) and “predominantly positive drinking attitudes” (scores ≥ 2.14).

#### 2.3.2. Alcohol-Related Problems

Alcohol-related problems were assessed using the 10-item Alcohol Use Disorders Identification Test (AUDIT) [3,64]. The AUDIT is a screening instrument used for measuring alcohol consumption and related problems, and it has been implemented in a wide range of settings and populations demonstrating measurement properties often superior to other alcohol-screening instruments [65]. Each item is scored in scale from 0 to 4, resulting in a sum score with a range of 0 to 40. Studies have supported the use of AUDIT as a unidimensional measure of alcohol-related problems [66], and a threshold of ≥8 scores has been recommended as an indication of alcohol-related problems [3,67]. The AUDIT demonstrated acceptable internal consistency (Cronbach’s *α* = 0.71). For the analyses, the sum score was dichotomized as recommended into two groups: employees with alcohol-related problems (score ≥ 8) and without them (scores 0–7).

#### 2.3.3. Moderators

Two variables were used as moderators in the study. Gender and Employment sector. Employment sector was constructed based on information about which branches (i.e., work divisions) the sample where employed. Branches were categorized using the European Classification of Economic Activities (Eurostat) [68], and further sorted into two groups of employment sectors: private-sector employees, which constituted the branches ‘transportation and storage’, ‘accommodation and food service activities’, and ‘manufacturing’; and public-sector employees, which constituted ‘public administration’, ‘education’, and ‘human health and social work activities’. Private companies with novation agreement from the public [69] (e.g., one company in human health and social work branch, which is part of the private sector but it is doing public duties) were included in the public sector employees’ group.

#### 2.3.4. Covariates

Based on prior research [70,71], age, gender, educational attainment, cohabitation status, occupational level (i.e., work position) and fraction of full-time work were considered potential confounders. To avoid over-adjustment, covariates were chosen based on a series of bivariate non-parametric correlation analyses (Spearman’s *rho*). A potential confounder was included as a covariate in adjusted analyses if (i) its bivariate association with the outcome (alcohol-related problems) displayed a *p*-value of <0.20, and (ii) it did not correlate highly (*rho* = ≤70) with another potential confounder [72]. Consequently, the following were included as covariates: *age* (18–29 years; 30–44 years; ≥45 years), *gender* (male; female), *cohabitation status* (living alone; living with others), *educational attainment* (primary/lower secondary; upper secondary; university/college), and *fraction of full-time work* (10–50%; >50–90%; 100%). 

### 2.4. Analysis

Employees’ drinking attitudes, stratified by gender, were explored using descriptive statistics. Frequencies (*n*) and proportions (%) for agreement/disagreement with each attitude item and for employees with predominantly negative/positive attitudes were computed; means (*M*) and standard deviations (*SD*) were calculated for the DNS scale. Gender differences were tested using bivariate chi-square tests of independence and adjusted one-way analysis of covariance (ANOVA), controlling for age, cohabitation status, and educational attainment. The prevalence of alcohol-related problems was the proportion of employees who scored ≥8 on the AUDIT. Differences in the prevalence of alcohol-related problems between employees with predominantly negative drinking attitudes versus employees with more positive attitudes were examined with chi-square tests of independence.

Multiple unconditional logistic regression models were built to obtain the odds ratios (OR), and corresponding 95% confidence intervals, of the association between drinking attitudes (predominantly negative versus positive) and alcohol-related problems for all employees, adjusted for age, gender, cohabitation status, educational attainment, fraction of full-time work, and employment sector (Model 1). An interaction term (continuous mean DNS scale score x gender) was included in Model 1 to determine whether gender moderated the association between drinking attitudes and alcohol problems. To determine whether the association varied by employment sector group, a two-way attitude variable × employment sector interaction was examined. Since the interaction with gender was statistically significant, we ran additional gender-stratified regression models (Model 2 for men, and Model 3 for women). To provide an indication of the amount of variation in alcohol-related problems explained by the model, the Cox & Snell R^2^ as well as the Nagelkerke R^2^ values were added to the model. All analyses were performed using IBM SPSS, Version 25, and statistical significance was set at *p* < 0.05.

### 2.5. Ethics

Participants were informed about the study’s aims and assured that their participation was voluntary. All participants provided written informed consent prior to participation and were informed told they could withdraw their consent at any given time without any consequences. The study was approved by the Regional Committees for Medical and Health Research in Norway (REK) (Reference Number 2014/647).

## 3. Results

### 3.1. Employees’ Attitudes toward Alcohol

Overall, a majority of the participants (61.5%) reported predominantly positive drinking attitudes. Table 2 shows that a higher proportion of men than women (68.2% versus 58.0%) reported predominantly positive drinking attitudes, and the mean attitude score was higher (*p* < 0.001) in men (*M* = 2.23; *SD* = 0.48) than in women (*M* = 2.10; *SD* = 0.44).

### 3.2. Employees’ Alcohol Problems and Attitudes toward Alcohol

Overall, one out of ten employees (10.9%) reported alcohol-related problems (men = 18.1%; women = 7.2%; *p* < 0.001) (Table 3). Alcohol-related problems were more prevalent (*p* <0.001) among those employees with predominantly positive drinking attitudes (15.4%), than among those with predominantly negative attitudes (3.7%).

For all employees (adjusted for gender, age [as a continuous variable], cohabitation status, educational attainment, fraction of full-time work [as a continuous variable], employment sector, and the interaction between drinking attitudes and gender; Table 4, Model 1), employees with predominantly positive drinking attitudes were almost three times as likely to report alcohol-related problems, compared to those with predominantly negative drinking attitudes (OR = 2.75; 95% CI: 2.00–3.76). Model 1 explained between 8.5% and 17.1% of the variation in alcohol-related problems (Cox & Snell *R*^2^ = 0.085; Nagelkerke *R*^2^ = 0.171). Gender moderated the association between drinking attitudes and alcohol-related problems (interaction term DNS x gender: OR = 3.52; 95% CI: 2.24–5.55), but employment sector did not (OR = 1.03; 95% CI: 0.90–1.17).

After adjusting for age, cohabitation status, educational attainment, fraction of full-time work, and employment sector, the association between drinking attitudes and alcohol-related problems was stronger for women (Table 4, Model 3: OR = 5.21; 95% CI: 3.34–8.15) than for men (Table 4, Model 2: OR = 3.10; 95% CI: 2.11–4.55). Additional models adjusting for age in the three categories shown in Table 1 did not result in any meaningfully different results than those presented in Table 4.

## 4. Discussion

### 4.1. Discussion of Main Findings

This study, conducted with a heterogeneous employee sample, aimed to explore whether there is an association between drinking attitudes and alcohol-related problems among workers, and if this association was moderated by gender and/or employment sector. Our main findings were as follows: (i) predominantly positive (i.e., liberal) drinking attitudes were much more frequent than negative attitudes, and much frequently in men than in women, (ii) one out of ten employees reported alcohol-related problems, and employees with predominantly positive drinking attitudes were almost three times as likely to report alcohol-related problems than those with predominantly negative attitudes, (iii) the association between drinking attitudes and alcohol-related problems was considerably stronger for women than it was for men, but (iv) there were no differences by employment sector (public vs. private employees).

Discovering a higher prevalence of positive drinking attitudes among employees was not surprising since alcohol consumption is deeply integrated in the larger society, as well as in the occupational domain. Employees are regularly exposed to alcohol in work-related settings, e.g., when bonding with colleagues after work hours, at employer-sponsored social events, during work-related travels, and while entertaining clients and business associates [8,73]. Employees develop normative assumptions about behaviors framed within the appropriate organization’s drinking culture. Such normative assumptions within a work-related setting can influence the employees’ beliefs and the level of engagement in a behavior [25,74]. As such, alcohol does play an important role in workplace and work-related rituals as a marker of social belonging to the work group [75]. Male employees reported more positive drinking attitudes than their female counterparts, a finding that is consistent with earlier studies on non-working populations [49,76]. However, prior studies were conducted in a culture where drinking alcohol by females was not so socially acceptable. These prior studies focused in the individual’s drinking attitudes regarding their reference group and not, as we did, the individual’s personal attitudes toward drinking. Estimating one’s perception of others’ attitudes towards alcohol drinking may be affected by misperception and over- or underestimate others’ beliefs and actual drinking behaviors [56,57,58,59]. Norms that apply to men also tend to be more supportive of alcohol consumption [32]. Although it has become more socially acceptable for women to drink [77], especially in countries where gender roles have gradually realigned and become more equal [78], men still consume alcohol more frequently and more heavily than women [18]. In fact, being male is identified as a significant predictor for risky drinking [14] and more specifically for binge drinking [79]. Such pointed differences could explain the found less favorable drinking attitude by women.

Our findings showed an association between drinking attitudes and alcohol-related problems among employees. This association is consistent with earlier research, which found that individuals with positive drinking attitudes tend to drink more than individuals with more negative attitudes towards drinking [42,43,44,45,46,47]. Attitudes generally predict behavior, in particular when attitudes remain stable over time [39]. Having favorable attitudes toward a behavior increases the likelihood of actually performing that behavior [37]. In fact, one out of ten employees reported alcohol-related problems, and these problems were more prevalent in men than in women, which is in agreement with earlier studies [14,33,80,81,82,83].

Although both the positive drinking attitudes and alcohol-related problems were more frequent in men than in women, in accordance with prior findings [41], we also found that the association between drinking attitudes and alcohol-related problems was stronger for women than for men. Our data, however, do not reveal the mechanisms behind this finding. It may be that drinking attitudes at work are much more important predictors of alcohol-related problems for women than for men. In addition, men’s drinking may be more affected by external social pressures and masculinity concerns [84], while women may be somewhat more sensitive to internal factors such as drinking expectancies [85]. Drinking norms have also traditionally been more strict for women than for men [32], and women may, therefore, be more mindful of their internalized norms (attitudes) to avoid potential social sanctions. Our finding is in contrast with some of prior studies that found stronger attitude-drinking association among men. But these earlier findings may have been affected by either an overrepresentation of males (72% male) [51], or by a culture whereby male drinking is more often tolerated than female drinking [49]. Further research is needed to disentangle the complex relationship between gender, drinking attitudes and health.

We were also interested in the role of the norms at different type of industries and branches in shaping one’s attitudes and behaviors toward drinking. Each work setting, based on job duties, position, and workload, may have unique cultural dimensions [25,30]. We, however, did not find differences by employment sector in the association between drinking attitudes and alcohol-related problems. Our finding is at odds with earlier studies, which reported differences by type of work setting [30,31,34,36,86]. However, our study was conducted in Norway and prior studies reported different traditional organizational cultures and regulations of drinking alcoholic beverages (e.g., drinking before or during work shifts) than those found in Norway. Further, Norway has a strict alcohol policy, and it is uncommon to find people working under the influence of alcohol in most Norwegian workplaces [87]. Excessive alcohol consumption can be regarded as a serious infringement of approved company regulations and norms [75], regardless of one’s occupation or industry setting. Still, external factors may become unwritten rules, including workers’ pre-existing attitudes and behaviors as well as cultural and social norms in the workers’ wider community. All these factors should be noted whenever considering the relationship between workplace and alcohol drinking patterns and the forming of attitudes and beliefs within a work culture [88]. Values and cultures can both be co-created through a process of socialization in a work setting as a set of shared understandings [89]. Differences in those factors could explain the disparities between our findings and prior studies. 

### 4.2. Methodological Issues

This study has several strengths. It was based on a large heterogeneous sample of employees, and it measured drinking attitudes and alcohol-related problems using validated instruments (i.e., the Drinking Norms Scale [31] and the Alcohol Use Disorders Identification Test [3,64]). However, there are methodological consideration to take into account when interpreting our findings. 

First, the cross-sectional nature of our study precludes drawing causal inferences about the relationships between social drinking attitudes and alcohol-related problems. The association between drinking attitudes and alcohol-related problems could be interpreted as attitudes leading to drinking behaviors and, subsequently, these to alcohol problems. But, as others have suggested, it may also be that behavior precedes attitudes [38], such as heavy drinking behaviors form more positive drinking attitudes. However, we think this explanation is not as likely as the assumption that attitudes precede behavior as mainstream health behavior models assume [40]. 

Second, although the sample for this study was relatively large (*N* = 4094), the response rate was low (23.0%). Lower response rates, however, are part of general declining participation rates in surveys [90]. Further, comparisons between the study sample and the target population (public and private salaried-employees in any blue, white or pink-collar occupations) indicated no differences in gender (*p* = 0.613) and only a few percent points of difference in the proportion of employees age ≤39, who were underrepresented in our sample (difference in percentage points = 4.9; *p* < 0.001). Thus, our analytical sample should be considered a fair representation of our target population. Compared with the composition of overall Norwegian workforce, our sample had an overrepresentation of women, employees age ≥40, employees with university/college education, and somewhat higher proportion of employees in the public/state sector. Nevertheless, our sample was not intended to represent the workforce of Norway so we caution generalizations of our findings to the Norway working population.

Third, all the data for this study was self-reported. As such, our results may have been affected by recall bias and social desirability. However, for some of our main variables of interest (i.e., attitudes), there’s no direct measurement alternative. Moreover, all data were collected using validated measurements instruments, with good reliability and validity. These instruments help ensure that the measures collected were in fact measuring what they were supposed to measure.

### 4.3. Implications

Findings from our study suggest that drinking attitudes should be considered when designing and conducting alcohol preventive interventions targeting employees. These interventions may target attitudes at the individual level or, perhaps better, at a group level addressing workplace drinking cultures. Attitudes are learned through socialization [91] and the socialization sources may be the various sociocultural levels to which individuals are exposed to. An important level may be the individual’s workplace. Intervention can be aimed to establish a “discouraging” workplace drinking culture, taking into account factors such as actual alcohol availability and workplace social control [25,30,31,32,92,93]. Emphasizing the role of drinking attitudes for interventions may be of particular importance for those workplaces where women are well represented, insofar that the actual association between drinking attitudes and alcohol-related problems may be stronger for women than for men.

Further research on the relationships between drinking attitudes and alcohol-related problems is definitely warranted. That effort would benefit from utilizing research designs that allow further exploration of development in study variables over time (e.g., prospective cohort studies), as well as by investigating a broader range of potential moderating and mediating variables.

## 5. Conclusions

Harmful alcohol consumption is indeed a major public health challenge, and drinking by employees is associated with detrimental occupational outcomes (e.g., absenteeism and presenteeism, that is, reduced on-the-job performance). This study highlights the role of drinking attitudes in alcohol-related problems among employees and that the impact of drinking attitudes on alcohol problems may vary across genders. The results of this study underscore the complexities that exist in the intersections between individual and sociocultural domains, and that attitudes should be emphasized for alcohol preventive interventions targeting employees.

## Figures and Tables

**Table 1:** Sample characteristics of all employees (*N* = 4094) and stratified by gender (men: *n* = 1398; women: *n* = 2696).

Variables	All Employees	Men	Women	
	*n* (%)	*n* (%)	*n* (%)	*p*-Value ^1^
**Age**				<0.001
18–29	422 (10.3)	127 (9.1)	295 (10.9)	
30–44	1440 (35.2)	469 (33.5)	971 (36.0)	
≥45	2232 (54.5)	802 (57.4)	1430 (53.0)	
**Cohabitation Status**				0.143
Living alone	589 (14.4)	204 (14.6)	385 (14.3)	
Living with others	3505 (85.6)	1194 (85.4)	2311 (85.7)	
**Educational Attainment**				<0.001
Primary/lower secondary	105 (2.6)	56 (4.0)	49 (1.8)	
Upper secondary	928 (22.7)	331 (23.7)	597 (22.1)	
University/college	3061 (74.7)	1011 (72.3)	2050 (76.0)	
**Fraction of full-time work**				0.001
10–50%	110 (2.7)	25 (1.8)	85 (3.2)	
>50–90%	663 (16.2)	97 (6.9)	566 (21.0)	
100%	3320 (81.1)	1276 (91.3)	2044 (75.8)	
**Employment sector**				<0.001
Private sector employees	394 (9.6)	310 (22.2)	84 (3.1)	
Public sector employees	3700 (90.4)	1088 (77.8)	2612 (96.9)	

^1^ Differences between men and women tested with chi-square tests of independence.

**Table 2:** Employees’ drinking attitudes, stratified by gender.

Drinking Attitudes	Men (*n* = 1398)		Women (*n* = 2696)		
	Disagree ^1^	Agree ^2^	Disagree ^1^	Agree ^2^	*p*-Value
	*n* (%)	*n* (%)	*n* (%)	*n* (%)	
**Drinking Norms Scale statements**					
**S1:** Having a drink or two at home after work is a harmless way to relax and unwind	917 (65.6)	481 (34.4)	1933 (71.1)	763 (28.3)	<0.001 ^5^
**S2:** Getting together for drinks once in a while after work with co-workers can improve employees’ morale	554 (39.6)	844 (60.4)	1396 (51.8)	1300 (48.2)	<0.001 ^5^
**S3:** Drinking with clients or customers is good for business	890 (63.7)	508 (36.3)	2191 (81.3)	505 (18.7)	<0.001 ^5^
**S4:** Supervisors miss key information if they don’t socialize with colleagues over a drink	1062 (76.0)	336 (24.0)	2354 (87.3)	342 (12.7)	<0.001 ^5^
**S5:** A drink or two a day is good for a person’s health	1059 (75.8)	339 (24.2)	2251 (83.5)	445 (16.5)	<0.001 ^5^
**S6 (Reversed score):** The more frequently people are exposed to alcohol, the more likely they are to develop a drinking problem	237 (17.0)	1161 (83.0)	623 (23.1)	2073 (76.9)	<0.001 ^5^
**S7 (Reversed score):** Serving alcohol at company social events sets a bad example for employees	982 (70.2)	416 (29.8)	1957 (72.6)	739 (27.4)	<0.001 ^5^
**Drinking Norms Scale (continuous scores) ^3^**					<0.001 ^6^
Mean (SD)	2.23 (0.48)		2.10 (0.44)		
**Drinking Norms Scale (dichotomized scores) ^4^**					<0.001 ^5^
Negative, *n* (%)	444 (31.8)		1131 (42.0)		
Positive, *n* (%)	954 (68.2)		1565 (58.0)		

^1^ Response categories “strongly disagree” and “disagree”; ^2^ Response categories “strongly agree” and “agree”; ^3^ Composite (mean) score of the seven Drinking Norms Scale items, potential range = 1–4, higher score indicates positive attitudes; ^4^ Dichotomization of mean scale score based on median split: negative < 2.14, positive = scores ≥ 2.14; ^5^ Gender differences tested with chi-square test of independence; ^6^ Differences tested using a one-way analysis of covariance (ANCOVA), adjusted for age, cohabitation status, and educational attainment.

**Table 3:** Alcohol-related problems by drinking attitudes.

	Drinking Attitudes ^1^		Total *n* (%)
Alcohol-Related Problems ^2^	Predominantly Negative *n* (%)	Predominantly Positive *n* (%)	
No	1517 (96.3)	2130 (84.6)	3647 (89.1)
Yes	58 (3.7)	389 (15.4)	447 (10.9)

^1^ Dichotomization of mean scale score based on median split: negative < 2.14, positive = scores ≥ 2.14; ^2^ Sum score, based on AUDIT—Alcohol Use Disorders Identification Test: scores 0–7 = No, scores 8–40 = Yes.

**Table 4:** Associations (OR and 95% CI) between drinking attitudes and alcohol-related problems, overall (Model 1) and stratified by gender (Models 2 and 3).

Variables		Model 1	Model 2	Model 3
		All Employees	Men	Women
		*n* = 4094	*n* = 1398	*n* = 2696
**Drinking Attitudes** (Positive vs. Negative [Ref.])	(OR_crude_)	(4.77)	(3.46)	(5.91)
	OR_adjusted_	2.75	3.1	5.21
	95% CI	2.00–3.76	2.11–4.55	3.34–8.15
	*p*-value	<0.001	<0.001	<0.001
**Gender** (female vs. male [Ref.])	OR_adjusted_	0.02		
	95% CI	0.01–0.07	-	-
	*p*-value	<0.001		
**Age** (in years)	OR_adjusted_	0.97	0.97	0.97
	95% CI	0.96–0.98	0.96–0.98	0.95–0.98
	*p*-value	<0.001	<0.001	<0.001
**Cohabitation Status** (Living with others vs. Living alone [Ref.])	OR_adjusted_	0.49	0.49	0.47
	95% CI	0.37–0.64	0.35–0.71	0.33–0.67
	*p*-value	<0.001	<0.001	<0.001
**Educational Attainment** (Upper secondary and University/college vs. Primary/lower secondary [Ref.])	OR_adjusted_	0.84	0.81	0.85
	95% CI	0.46–1.54	0.40–1.62	0.24–2.97
	*p*-value	0.58	0.56	0.8
	OR_adjusted_	0.71	0.63	0.8
	95% CI	0.39–1.31	0.31–1.28	0.23–2.77
	*p*-value	0.28	0.2	0.73
**Fraction of full-time work** (in percent)	OR_adjusted_	1	1.01	0.99
	95% CI	0.99–1.01	0.99–1.02	0.99–1.00
	*p*-value	0.62	0.14	0.69
**Employment Sector** (Public vs. Private employees [Ref.])	OR_adjusted_	0.71	0.76	0.59
	95% CI	0.52–0.97	0.52–1.11	0.30–1.12
	*p*-value	<0.05	0.16	0.11
**Interaction attitudes x Gender**	OR_adjusted_	3.3	-	-
	95% CI	2.10–5.21		
	*p*-value	<0.001		
Cox & Snell *R*^2^		0.085	0.071	0.049
Nagelkerke *R*^2^		0.171	0.116	0.122

OR_crude_ = odds ratio, bivariate association; OR_adjusted_ = adjusted OR for the other variables included in the model; CI = 95% confidence intervals. Ref. = Reference category.

## References

[1] World Health Organization (2018). Global Status Report on Alcohol and Health 2018.

[2] Griswold M.G., Fullman N., Hawley C., Arian N., Zimsen S.R.M., Tymeson H.D., Venkateswaran V., Tapp A.D., Forouzanfar M.H., Salama J.S. (2018). Alcohol use and burden for 195 countries and territories, 1990–2016: A systematic analysis for the Global Burden of Disease Study 2016. Lancet.

[3] Babor T.F., Higgins-Biddle J.C., Saunders J.B., Monteiro M.G. (2001). AUDIT: The Alcohol Use Disorders Identification Test. Guidelines for Use in Primary Health Care.

[4] Anderson P., Baumberg B. (2006). Alcohol in Europe—Public Health perspective: Report summary. Drugs Educ. Prev. Policy.

[5] Baumberg B. (2006). The global economic burden of alcohol: A review and some suggestions. Drug Alcohol Rev..

[6] Collin J., Casswell S. (2016). Alcohol and the sustainable development goals. Lancet.

[7] Gordon R., Heim D., MacAskill S. (2012). Rethinking drinking cultures: A review of drinking cultures and a reconstructed dimensional approach. Public Health.

[8] Nordaune K., Skarpaas L.S., Sagvaag H., Haveraaen L., Rimstad S., Kinn L.G., Aas R.W. (2017). Who initiates and organises situations for work-related alcohol use? The WIRUS culture study. Scand. J. Public Health.

[9] Schou L., Moan I.S. (2016). Alcohol use–sickness absence association and the moderating role of gender and socioeconomic status: A literature review. Drug Alcohol Rev..

[10] Thørrisen M.M., Bonsaksen T., Hashemi N., Kjeken I., van Mechelen W., Aas R.W. (2019). Association between alcohol consumption and impaired work performance (presenteeism): A systematic review. BMJ Open.

[11] Roman P.M., Blum T.C. (2002). The workplace and alcohol problem prevention. Alcohol Res. Health.

[12] Frone M.R. (2013). Alcohol and Illicit Drug Use in the Workforce and Workplace.

[13] Moan I.S., Halkjelsvik T. (2016). Alkohol og arbeidsliv. En Undersøkelse Blant Norske Arbeidstakere [Alcohol and Work. A Survey among Norwegian Employees].

[14] Thørrisen M.M., Skogen J.C., Aas R.W. (2018). The associations between employees’ risky drinking and sociodemographics, and implications for intervention needs. BMC Public Health.

[15] Ames G.M., Bennett J.B. (2011). Prevention interventions of alcohol problems in the workplace. Alcohol Res. Health.

[16] Brady K.T., Back S.E., Greenfield S.F. (2009). Women and Addiction: A Comprehensive Handbook.

[17] Wilsnack R.W., Vogeltanz N.D., Wilsnack S.C., Harris R. (2000). Gender differences in alcohol consumption and adverse drinking consequences: Cross-cultural patterns. Addiction.

[18] Wilsnack R.W., Wilsnack S.C., Kristjanson A.F., Vogeltanz-Holm N.D., Gmel G. (2009). Gender and alcohol consumption: Patterns from the multinational GENACIS project. Addiction.

[19] Rehm J., Room R., Graham K., Monteiro M., Gmel G., Sempos C.T. (2003). The relationship of average volume of alcohol consumption and patterns of drinking to burden of disease: An overview. Addiction.

[20] Kuntsche S., Gmel G., Knibbe R.A., Kuendig H., Bloomfield K., Kramer S., Grittner U. (2006). Gender and cultural differences in the association between family roles, social stratification, and alcohol use: A European cross-cultural analysis. Alcohol Alcohol..

[21] Mäkelä K., Gmel G., Grittner U., Kuendig H., Kuntsche S., Bloomfield K., Room R. (2006). Drinking patterns and their gender differences in Europe. Alcohol Alcohol..

[22] Bratberg G.H., Wilsnack S.C., Wilsnack R., Haugland S.H., Krokstad S., Sund E.R., Bjørngaard H. (2016). Gender differences and gender convergence in alcohol use over the past three decades (1984–2008), The HUNT study, Norway. BMC Public Health.

[23] Chirkov V. (2019). An introduction to the theory of sociocultural models. Asian J. Soc. Psychol..

[24] Savic M., Room R., Mugavin J., Pennay A., Livingston M. (2016). Defining "drinking culture": A critical review of its meaning and connotation in social research on alcohol problems. Drugs Educ. Prev. Policy.

[25] Ames G.M., Janes C. (1992). A cultural approach to conceptualizing alcohol and the workplace. Alcohol Health Res. World.

[26] Makela K. (1986). Attitudes towards drinking and drunkeness in four Scandinavian countries. Ann. N. Y. Acad. Sci..

[27] Schulte M.T., Ramo D., Brown S.A. (2009). Gender differences in factors influencing alcohol use and drinking progression among adolescents. Clin. Psychol Rev..

[28] Monk R.L., Heim D. (2014). A systematic review of the Alcohol norms literature: A focus on context. Drugs Educ. Prev. Policy.

[29] Room R., Mitchell A. (1972). Notes on cross-national and cross-cultural studies. Drink. Drug Pract. Surv..

[30] Ames G.M., Grube J.W., Moore R.S. (2000). Social control and workplace drinking norms: A comparison of two organizational cultures. J. Stud. Alcohol.

[31] Barrientos-Gutierrez T., Gimeno D., Mangione T.W., Harrist R.B., Amick B.C. (2007). Drinking social norms and drinking behaviours: A multilevel analysis of 137 workgroups in 16 worksites. Occup. Environ. Med..

[32] Hodgins D.C., Williams R., Munro G. (2009). Workplace responsibility, stress, alcohol availability and norms as predictors of alcohol consumption-related problems among employed workers. Substance Use Misuse.

[33] Kjærheim K., Mykletun R., Aasland O.G., Haldorsen T., Andersen A. (1995). Heavy drinking in the restaurant business: The role of social modelling and structural factors of the work-place. Addiction.

[34] Ames G.M., Duke M.R., Moore R.S., Cunradi C.B. (2009). The Impact of Occupational Culture on Drinking Behavior of Young Adults in the US Navy. J Mix Method Res..

[35] Moan I.S., Halkjelsvik T. (2019). Alkohol og Arbeidsliv II. Bruk, Konsekvenser og Retningslinjer Ved Ulike Typer Arbeidsplasser i Norge [Alcohol and Work life II. Use, Consequences and Guidelines at Different Types of Workplaces in Norway].

[36] Linsky A.S., Colby J.P., Straus M.A. (1986). Drinking norms and alcohol-related problems in the United States. J. Stud. Alcohol.

[37] Ajzen I. (1991). The theory of planned behavior. Organ. Behav. Hum. Decis. Process..

[38] Bem D.J. (1967). Self-perception: An alternative interpretation of cognitive dissonance phenomena. Psychol. Rev..

[39] Glasman L.R., Albarracin D. (2006). Forming attitudes that predict future behavior: A meta-analysis of the attitude-behavior relation. Psychol. Bull..

[40] Armitage C.J., Conner M. (2000). Social cognition models and health behaviour: A structured review. Psychol. Health.

[41] Cooke R., Dahdah M., Norman P., French D.P. (2016). How well does the theory of planned behaviour predict alcohol consumption? A systematic review and meta-analysis. Health Psychol. Rev..

[42] Foxcroft D.R., Lister-Sharp D., Lowe G. (1997). Alcohol misuse prevention for young people: A systematic review reveals methodological concerns and lack of reliable evidence of effectiveness. Addiction.

[43] Mcalaney J., McMahon J. (2007). Normative beliefs, misperceptions, and heavy episodic drinking in a British student sample. J. Stud. Alcohol Drugs.

[44] McCarty D., Morrison S., Mills K.C. (1983). Attitudes, beliefs and alcohol use. An. analysis of relationships. J. Stud. Alcohol.

[45] Morgenstern M. (2011). Attitudes as mediators of the longitudinal association between alcohol advertising and youth drinking. Arch. Pediatr. Adolesc. Med..

[46] Stacy A.W., Bentler P.M., Flay B.R. (1994). Attitudes and health behavior in diverse populations: Drunk driving, alcohol use, binge eating, marijuana use, and cigarette use. Health Psychol..

[47] Treno A.J., Alaniz M.L., Gruenewald P.J. (2000). The use of drinking places by gender, age and ethnic groups: An analysis of routine drinking activities. Addiction.

[48] Wall A.M., Hinson R.E., McKee S.A. (1998). Alcohol outcome expectancies, attitudes toward drinking and the theory of planned behavior. J. Stud. Alcohol.

[49] Kirmani M.N., Suman L.N. (2010). Gender differences in alcohol related attitudes and expectancies among college students. J. Indian Acad. Appl. Psychol..

[50] Conner M., Warren R., Close S., Sparks P. (1999). Alcohol consumption and the theory of planned behavior: An examination of the cognitive mediation of past behavior. J. Appl Soc. Psychol..

[51] DiBello A.M., Miller M.B., Neighbors C., Reid A., Carey K.B. (2018). The relative strength of attitudes versus perceived drinking norms as predictors of alcohol use. Addict. Behav..

[52] Gibbons F.X., Gerrard M. (1995). Predicting young adults’ health risk behavior. J. Pers Soc. Psychol..

[53] Kuther T.L. (2002). Rational decision perspectives on alcohol consumption by youth. Revising the theory of planned behavior. Addict. Behav..

[54] McEachan R.R.C., Conner M., Taylor N.J., Lawton R.J. (2011). Prospective prediction of health-related behaviours with the theory of planned behaviour: A meta-analysis. Health Psychol. Rev..

[55] McMillan B., Conner M. (2003). Using the theory of planned behaviour to understand alcohol and tobacco use in students. Psychol. Health Med..

[56] Amialchuk A., Ajilore O., Egan K. (2019). The influence of misperceptions about social norms on substance use among school-aged adolescents. Health Econ..

[57] Perkins H., Xenitidou M., Edmonds B. (2014). Misperception Is Reality: The “Reign of Error” About Peer Risk Behaviour Norms Among Youth and Young Adults. The Complexity of Social Norms. Computational Social Sciences.

[58] Prentice D.A., Miller D.T. (1993). Pluralistic Ignorance and Alcohol-Use on Campus—Some Consequences of Misperceiving the Social Norm. J. Pers. Soc. Psychol.

[59] Borsari B., Carey K.B. (2003). Descriptive and injunctive norms in college drinking: A meta-analytic integration. J. Stud. Alcohol.

[60] Franca L.R., Dautzenberg B., Reynaud M. (2010). Heavy Episodic Drinking and Alcohol Consumption in French Colleges: The Role of Perceived Social Norms. Alcoholism (NY).

[61] Andersson A., Wiréhn A.-B., Ölvander C., Ekman D.S., Bendtsen P. (2009). Alcohol use among university students in Sweden measured by an electronic screening instrument. BMC Public Health..

[62] Clements R. (1999). Prevalence of alcohol-use disorders and alcohol-related problems in a college student sample. J. Am. Coll Health.

[63] Lewis M.A., Neighbors C. (2006). Social norms approaches using descriptive drinking norms education: A review of the research on personalized normative feedback. J. Am. Coll. Health.

[64] Saunders J.B., Aasland O.G., Babor T.F., de la Fuente J.R., Grant M. (1993). Development of the Alcohol Use Disorders Identification Test (AUDIT): WHO collaborative project on early detection of persons with harmful alcohol consumption-II. Addiction.

[65] de Meneses-Gaya C., Zuardi A.W., Loureiro S.R., Crippa J.A.S. (2009). Alcohol Use Disorders Identification Test (AUDIT): An updated systematic review of psychometric properties. Psychol. Neurosci..

[66] Skogen J.C., Thorrisen M.M., Olsen E., Hesse M., Aas R.W. (2019). Evidence for essential unidimensionality of AUDIT and measurement invariance across gender, age and education. Results from the WIRUS study. Drug Alcohol Depen..

[67] Conigrave K.M., Hall W.D., Saunders J.B. (1995). The AUDIT questionnaire: Choosing a cut-off score. Addiction.

[68] NACE Rev. 2 (2008). Statistical Classification of Economic Activities in the European Community.

[69] Eidsheim L.H. (2007). Assignment of Contractual Rights: Legal and Linguistic Challenges.

[70] Li J., Wu B., Selbæk G., Krokstad S., Helvik A.S. (2017). Factors associated with consumption of alcohol in older adults—A comparison between two cultures, China and Norway: The CLHLS and the HUNT-study. BMC Geriatr..

[71] Murphy A., Roberts B., Stickley A., McKee M. (2012). Social factors associated with alcohol consumption in the former Soviet Union: A systematic review. Alcohol Alcohol..

[72] Hosmer D.W., Lemeshow S., Sturdivant R.X. (2013). Applied Logistic Regression.

[73] Nesvåg S., Ramvi E., Tungland E.M. (1999). Arbeidslivet i forandring: Nye rammer for alkoholbruk. Nord. Stud. Alcohol Drugs.

[74] Bacharach S.B., Bamberger P.A., Sonnenstuhl W.J. (2002). Driven to drink: Managerial control, work-related risk factors, and employee problem drinking. Acad. Manag. J..

[75] Nesvåg S., Duckert F. (2017). Work-related drinking and processes of social integration and marginalization in two Norwegian workplaces. Cult. Organ..

[76] Sukhwal M., Suman L.N. (2008). Alcohol related beliefs among college students. Indian J. Clin. Psychol..

[77] Bühringer G. (2006). Germany, alcohol and alcohol policy: Oscilliating between contemplation, action and relapse. Addiction.

[78] Rahav G., Wilsnack R., Bloomfield K., Gmel G., Kuntsche S. (2006). The influence of societal level factors on men’s and women’s alcohol consumption and alcohol problems. Alcohol Alcohol..

[79] Naimi T.S., Nelson D.E., Brewer R.D. (2010). The intensity of binge alcohol consumption among U.S. adults. Am. J. Prev. Med..

[80] Howland J., Mangione T., Kuhlthau K., Bell N., Heeren T., Lee M., Levine S. (1996). Work-site variation in managerial drinking. Addiction.

[81] Kawakami N., Haratani T., Hemmi T., Araki S. (1992). Prevalence and demographic correlates of alcohol-related problems in Japanese employees. Soc. Psychiatry Psychiatr. Epidemiol..

[82] Marchand A., Parent-Lamarche A., Blanc M.E. (2011). Work and High-Risk Alcohol Consumption in the Canadian Workforce. Int. J. Environ. Res. Public Health.

[83] Webb G.R., Redman S., Hennrikus D., Rostas J.A.P., Sanson-Fisher R.W. (1990). The prevalence and sociodemographic correlates of high-risk and problem drinking at an industrial worksite. Br. J. Addiction.

[84] de Visser R.O., McDonnell E.J. (2013). “Man points”: Masculine capital and young men’s health. Health Psychol..

[85] Guise J.M.F., Gill J.S. (2006). ‘Binge drinking? It’s good, it’s harmless fun’: A discourse analysis of accounts of female undergraduate drinking in Scotland. Health Educ. Res..

[86] Yang M.J., Yang M.S., Kawachi I. (2001). Work experience and drinking behavior: Alienation, occupational status, workplace drinking subculture and problem drinking. Public Health.

[87] Gjerde H., Christophersen A.S., Moan I.S., Yttreda L.B., Walsh J.M., Normann P.T., Mørland J. (2010). Use of alcohol and drugs by Norwegian employees: A pilot study using questionnaires and analysis of oral fluid. J. Occup. Med. Toxicol..

[88] Pidd K., Roche A.M., Moore D., Dietze P. (2008). Changing workplace cultures: An integrated model for the prevention and treatment of alcohol-related problems. Drugs and Public Health.

[89] Bochner S., O’Driscoll M., Taylor P., Kalliath T. (2003). Organisational culture and climate. Organisational Psychology in Australia and New Zealand.

[90] Czajka J.L., Beyler A. (2016). Declining Response Rates in Federal Surveys: Trends and Implications.

[91] Fishbein M., Ajzen I. (1975). Belief, Attitude, Intention and Behavior: An Introduction to Theory and Research.

[92] Frone M.R., Trinidad J.R. (2012). Testing a general model of employee alcohol use and workplace productivity among U.S. workers. Alcohol. Clin. Exp. Res..

[93] Moore R.S., Ames G.M., Duke M.R., Cunradi C.B. (2012). Food service employee alcohol use, hangovers and norms during and after work hours. J. Subst. Use.

